# Organ Dose and Attributable Cancer Risk in Lung Cancer Screening with Low-Dose Computed Tomography

**DOI:** 10.1371/journal.pone.0155722

**Published:** 2016-05-20

**Authors:** Natalia Saltybaeva, Katharina Martini, Thomas Frauenfelder, Hatem Alkadhi

**Affiliations:** Institute for Diagnostic and Interventional Radiology, University Hospital Zurich, University of Zurich, Zurich, Switzerland; Universität Bochum, GERMANY

## Abstract

**Purpose:**

Lung cancer screening with CT has been recently recommended for decreasing lung cancer mortality. The radiation dose of CT, however, must be kept as low as reasonably achievable for reducing potential stochastic risks from ionizing radiation. The purpose of this study was to calculate individual patients’ lung doses and to estimate cancer risks in low-dose CT (LDCT) in comparison with a standard dose CT (SDCT) protocol.

**Materials and Methods:**

This study included 47 adult patients (mean age 63.0 ± 5.7 years) undergoing chest CT on a third-generation dual-source scanner. 23/47 patients (49%) had a non-enhanced chest SDCT, 24 patients (51%) underwent LDCT at 100 kVp with spectral shaping at a dose equivalent to a chest x-ray. 3D-dose distributions were obtained from Monte Carlo simulations for each patient, taking into account their body size and individual CT protocol. Based on the dose distributions, patient-specific lung doses were calculated and relative cancer risk was estimated according to BEIR VII recommendations.

**Results:**

As compared to SDCT, the LDCT protocol allowed for significant organ dose and cancer risk reductions (p<0.001). On average, lung dose was reduced from 7.7 mGy to 0.3 mGy when using LDCT, which was associated with lowering of the cancer risk from 8.6 to 0.35 per 100’000 cases. A strong linear correlation between lung dose and patient effective diameter was found for both protocols (R^2^ = 0.72 and R^2^ = 0.75 for SDCT and LDCT, respectively).

**Conclusion:**

Use of a LDCT protocol for chest CT with a dose equivalent to a chest x-ray allows for significant lung dose and cancer risk reduction from ionizing radiation.

## Introduction

During the past decade, several studies focused on low-dose computed tomography (LDCT)-based screening for lung cancer [1–5]. According to the National Lung Screening Trial (NLST), lung cancer screening with LDCT has shown a survival benefit with a 20% reduction in lung cancer related mortality [6, 7]. However, there is an ongoing debate related to the radiation dose of LDCT and its associated potential risk of radiation-induced lung cancer. This is particularly important for screening programs due to the cumulative dose associated with repeated CT examinations in large cohorts of individuals.

The majority of the LDCT protocols used in current lung cancer screening trials were mainly achieved by reducing the tube current down to 40 mAs, resulting in estimated effective doses of 1–1.5mSv per CT examination [8–10]. Third-generation dual-source CT is equipped with a selective photon shield that eliminates lower energy photons from the x-ray spectrum, thus allowing for further radiation dose reduction[11]. Recent studies have shown that the estimated effective dose from chest CT can thus be reduced down to 0.06 mSv, being at the level of conventional chest x-ray while maintaining a good image quality of the examination [11–15]. In all these studies effective doses were estimated based on volume CT dose index (CTDI_vol_) values taken from the electronically logged protocols. Although this approach allows for comparison between different CT protocols, the CTDI_vol_ reflects the dose in uniform phantoms and cannot be used as the patient-specific dose [16, 17]. A more accurate assessment requires organ dose calculations taking the individual patient body habitus into account. In addition, the individual organ dose is also a better measure for estimating patient risk, because the effective dose is intended for estimating the radiation exposure of entire populations and not for individuals [17–19].

The aim of this study was to calculate individual patient lung doses and lifetime attributable risk of lung cancer from a LDCT protocol of the chest at a radiation dose equivalent to that of a chest x-ray and to compare these values with those from a standard chest CT protocol. Since lung cancer screening was recommended for individuals between the age of 55 and 74 years [10, 20], we focused our study explicitly on patients in this age group.

## Materials and Methods

### Patient population

The study included 47 consecutive patients between 55 and 74 years of age (mean age 63.0 ± 5.7 years; 27 males, mean age 64.0 ± 5.8 years and 20 females, mean age 63.0 ± 6.9 years) who were referred to our department for non-enhanced chest CT. Mean body mass index (BMI) was 26.3±5.6 kg/m^2^. Indications for chest CT were as follows: diffuse parenchymal lung disease (n = 23); this group underwent imaging with our standard dose CT (SDCT) protocol, and follow-up of known pulmonary nodules (n = 15) and suspicion of pulmonary infection in immunodeficient patients (n = 9); the latter patients (total n = 24) underwent imaging with our LDCT protocol described below.

This study had local institutional review board approval (Kantonale Ethikkommission Zürich) for retrospective use of CT images, from which identifying information has been removed. Written informed consent was waived because of the retrospective nature of the study.

### CT scanning and reconstruction

All patients were scanned cranio-caudally on a third-generation 192-slice dual-source CT scanner (SOMATOM Force, Siemens Healthcare, Forchheim, Germany) operated in the single-source mode.

Twenty-four of the 47 patients (51%) were scanned with the SDCT protocol with automatic attenuation-based tube voltage selection (CAREkV, Siemens) and attenuation-based tube current modulation (CareDose4D, Siemens) with quality reference values of 120 kVp and 70 mAs. The other twenty-three patients (49%) were scanned with LDCT at 100 kVp and with a quality reference tube current-time product of 45 mAs, as previously shown [11, 13, 15]. A 0.6 mm Sn-filter was used for spectral shaping by eliminating low energy photons from the spectrum. A pitch of 1.2, collimation of 0.6×96 mm using the z-flying focal spot, and a gantry rotation time of 0.5 seconds were used for both protocols. All CT images were reconstructed using a slice thickness of 2 mm, an increment of 1.6 mm, and using a sharp tissue convolution kernel (Bl64) with advanced modeled IR (ADMIRE) at a strength level of 3, as previously shown [13].

### Image quality analysis

One reader (K.M., with two years of experience in radiology), not involved in qualitative analysis, placed five circular region of interests (ROIs) in the subcutaneous fat tissue of each patient scanned with both SD and LDCT. The ROI size was fixed at 380 mm^2^. Average image noise was defined as the standard deviation of attenuation in consecutive ROIs at different slice positions, as previously shown elsewhere [13].

For both patient groups, subjective image quality (diagnostic vs. non-diagnostic) was evaluated by two other, independent readers (H.A., with 15 years of experience in radiology, T.F. with 14 years of experience in radiology).

### Monte Carlo simulations

Monte Carlo (MC) simulations were performed by using a commercially available software tool (ImpactMC, CT Imaging GmbH, Erlangen, Germany) to obtain 3D dose distributions. The accuracy of the tool was previously validated in anthropomorphic phantoms for both axial and spiral scanning modes [21]. MC simulations were performed using specific scanner geometry, filtration, collimation and tube voltage as used for the respective CT examinations. Individual patient images acquired from CT scanner were used as an input volume for MC simulations. The original tube current modulation curves together with start and end angular position of the tube were extracted from the raw data using a manufacturer-provided tool.

### Organ dose calculation

Based on a 3D dose distribution, any organ dose within the volume can be calculated as a mean value of all voxels assigned to a given organ. The voxels associated with lung tissue were identified based on the CT data explicitly for each patient by using global HU-based thresholding (ImageJ software). Then the lungs dose was calculated from the 3D dose distribution as a mean dose value within the segmented volume.

In order to investigate the organ dose dependency on patient size, we measured the individual patients’ lateral diameter D_lat_ and the antero-posterio diameter D_ap_ using their CT images. The effective diameter D_eff_ was calculated as follows:

$$D_{eff}=\;\sqrt{D_{lat}\times D_{ap}}$$ (1)

and was used as an indicator of patient size.

### Risk assessment

To estimate the potential risk of radiation-induced lung cancer we used a model proposed by BEIR VII [22]. This model was designed for estimating the lifetime attributable risk of an exposed individual developing cancer. According to BEIR VII the risk can be calculated as follows:

$$R_{n}=\frac{D_{n}}{0.1}\times k_{n}^{a,g}$$ (2)

R_n_ is the number of cancer cases per 100’000 persons for a specific organ n; D_n_ is the organ dose in Gy, and k_n_^a,g^ is an age- and gender-specific risk coefficient for organ n. The coefficients k_n_^a,g^ are tabulated in the BEIR VII report for males and females at discrete ages of 0, 5, 10, 15, 20, 30, 40, 50, 60, 70 and 80 years. Values of the risk coefficient at intermediate ages were determined by using linear interpolation. Since the individual risk depends not only on the organ dose but also on patient age and gender (see Eq 2), patients were divided into 3 age groups (55–60 years, 61–67 years, and 68–74 years). The average risk values within each age group were calculated separately for male and female patients in both LDCT and SDCT cohorts.

### Data analysis

All statistical analyses were performed using commercially available software (SPSS, release 22.0; SPSS, Chicago, IL, USA). Normal distribution was tested using the Shapiro-Wilk test. Post-hoc power analysis showed that a sample size of 24 in each patient group will provide a significant Pearson correlation with a medium effect size at a 0.05 level of significance and a power of 80%. Linear regression analysis was performed to assess the correlation between lung dose and patient effective diameter for both SDCT and LDCT protocols. The Student *t*-test for independent samples was performed to determine the significance of the differences in lung doses in LDCT and SDCT chest protocols. The same *t*-test was applied to show the significant difference in risk values estimated for LDCT protocol compared to SDCT one. A two-tailed *p-*value below 0.05 was considered to indicate a statistically significant difference.

## Results

**Fig 1:**
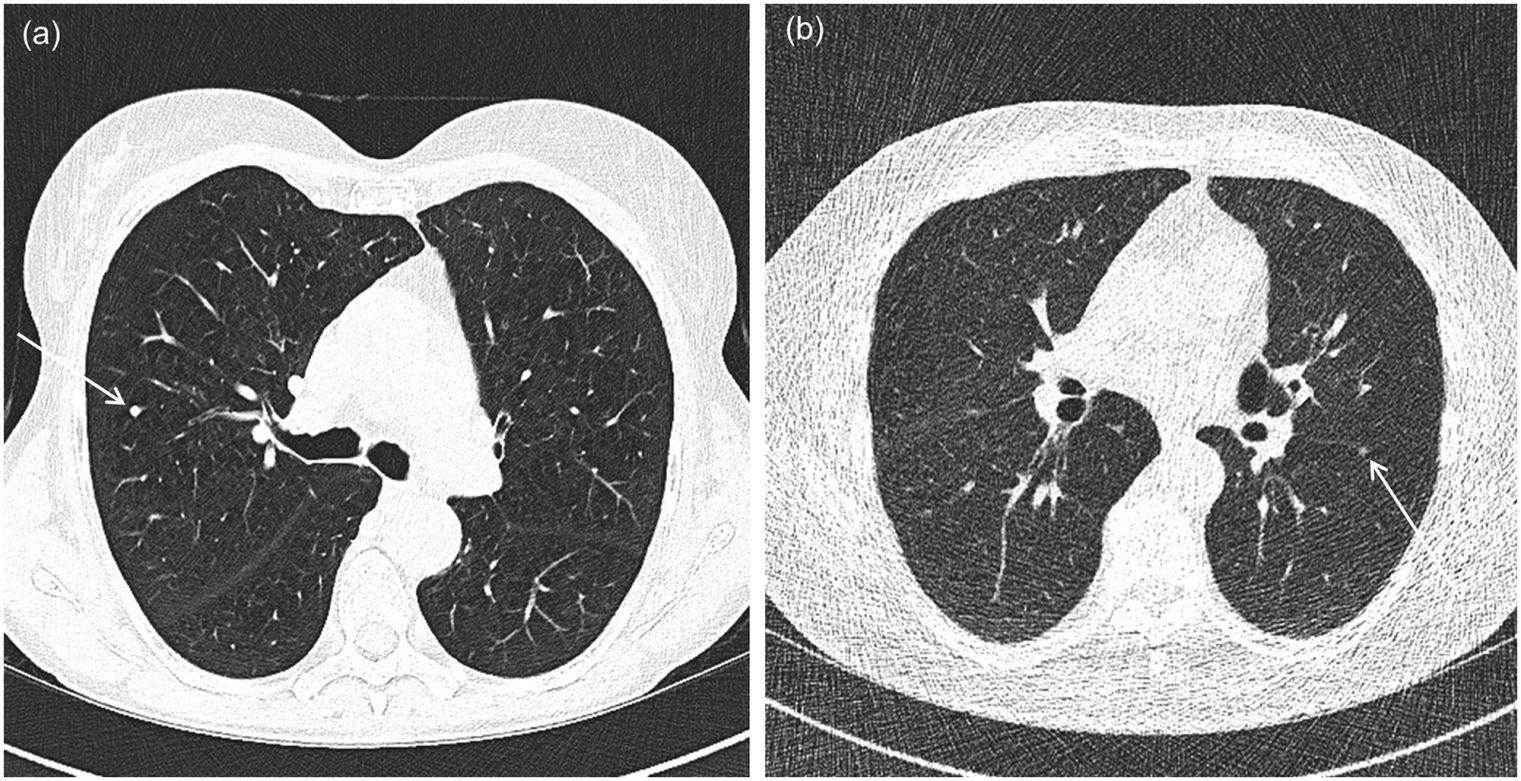
Representative transverse CT sections of a female patient with a nodule (arrow) scanned with a standard dose protocol (lung dose 5.6 mGy) (a), and a male patient with a ground glass nodule at the fissure (arrow) scanned with a low-dose CT protocol (lung dose 0.44 mGy) (b).

Representative image examples of two patients scanned with SDCT and with LDCT are provided in Fig 1. The lung dose for the patient scanned with the SDCT protocol equaled 5.6 mGy, while the lung dose for the patient scanned with LDCT was 0.44 mGy. Both CT examinations were considered to be of diagnostic image quality.

**Fig 2:**
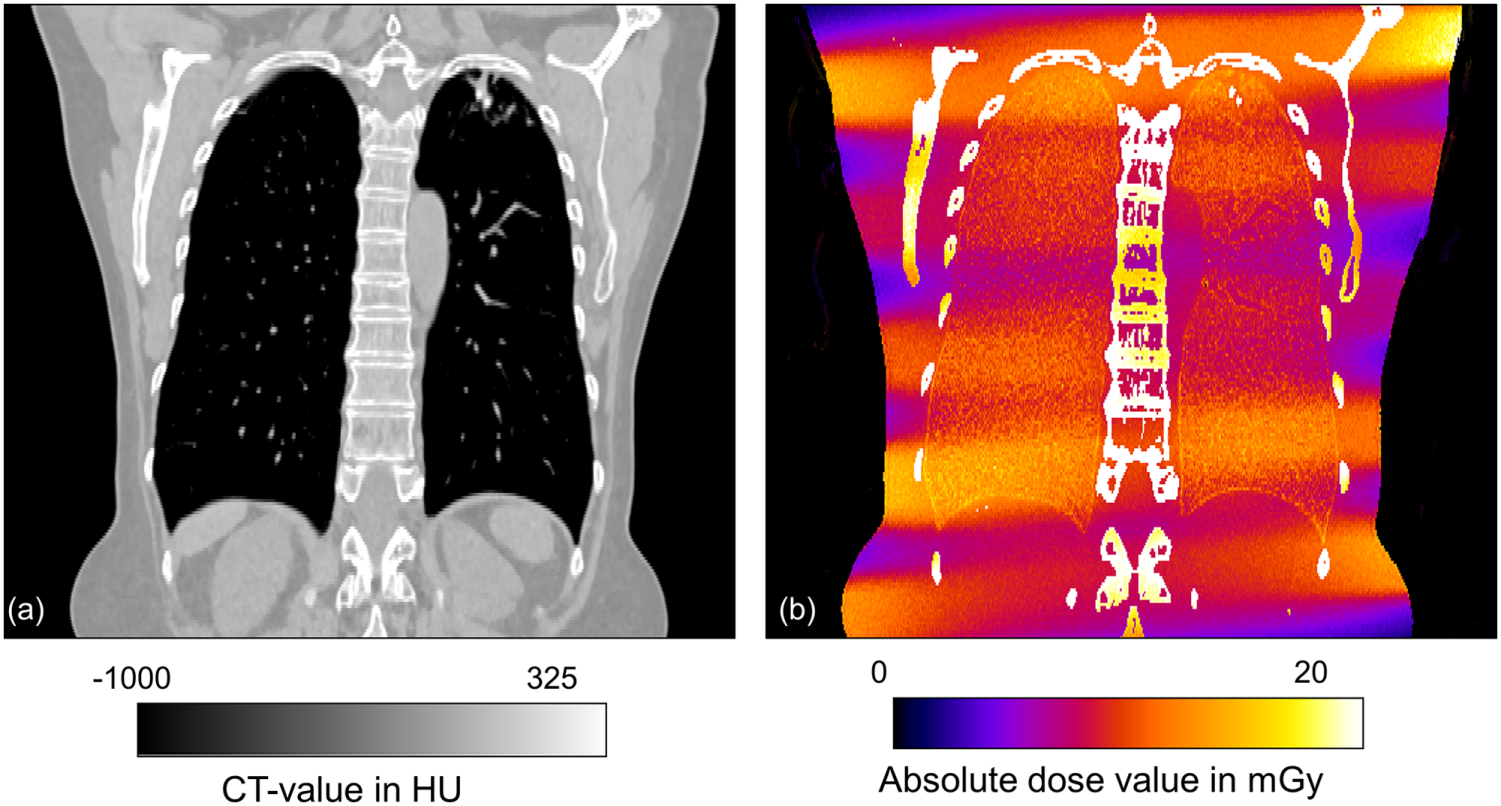
Coronal reformation of the CT for female patient (a) and corresponding dose distribution obtained by Monte Carlo simulations (b). Note the spiral trajectory of the x-ray tube leading to heterogeneous dose distributions.

Fig 2 shows an example of patient CT data and respective 3D dose distribution within the patient volume obtained by MC simulations. It can be seen that the spiral trajectory of the x-ray tube with a pitch of 1.2 is reflected by spiral stripes in dose distribution.

### Organ dose

Tables 1 and 2 show the patient age, gender, effective diameter, and the lung dose calculated from MC simulations for SDCT and LDCT, respectively. Both SDCT and LDCT patient populations categorized by age and effective diameter were normally distributed. The average lung dose was 7.7 mGy (range 4.9–10 mGy) and 0.3 mGy (range 0.16–0.52 mGy) for SDCT and LDCT, respectively.

**Table 1:** Dose and relative cancer risk for each individual patient (male-M, female-F) calculated for the standard dose protocol, sorted by patient age.

Standard dose protocol				
Patient gender	Patient age, [years]	Patient effective diameter, [cm]	Lung dose, [mGy]	Risk, [cases/ 10^5^]
F	55	27.20	8.20	17.67
M	55	30.85	9.00	8.55
F	56	22.65	4.89	10.40
M	58	32.03	9.20	8.30
M	59	31.75	8.20	7.49
M	60	32.65	8.60	7.65
M	62	24.49	6.50	5.47
F	63	30.50	9.30	17.19
F	63	26.27	6.65	12.29
M	64	27.48	8.77	6.96
M	64	23.24	5.33	4.23
M	64	31.19	7.90	6.27
F	65	25.10	5.60	9.74
M	65	28.55	9.30	7.16
F	67	27.71	7.20	11.75
M	68	24.68	5.70	3.98
M	68	30.82	8.40	5.86
F	69	25.69	7.74	11.80
F	69	27.53	8.30	12.65
M	71	34.00	9.04	5.60
M	72	36.00	10.00	5.88
M	74	29.91	7.82	4.11
F	76	25.92	6.70	7.97
**Mean**	**64.65**	**28.53**	**7.75**	**8.65**
**Maximum**	**76.00**	**36.00**	**10.00**	**17.67**
**Minimum**	**55.00**	**22.65**	**4.89**	**3.98**

**Table 2:** Dose and relative cancer risk for each individual patient (male-M, female-F) calculated for the low dose protocol, sorted by patient age.

Ultra-low dose protocol				
Patient gender	Patient age, [years]	Patient effective diameter, [cm]	Lung dose, [mGy]	Risk, [cases/ 10^5^]
F	55	28.91	0.299	0.64
F	55	24.6	0.23	0.50
M	56	34.07	0.45	0.42
M	56	32.86	0.4136	0.39
F	57	23.8	0.17	0.36
M	58	30.20	0.44	0.40
M	59	30.8	0.35	0.32
M	59	30.6	0.26	0.23
M	60	30.0	0.43	0.38
F	61	26.27	0.2	0.39
F	61	24.90	0.18	0.35
M	61	25.0	0.16	0.14
F	64	35.0	0.52	0.93
M	65	32.0	0.32	0.25
F	65	26.0	0.16	0.28
F	66	23.66	0.19	0.32
M	66	30.0	0.32	0.24
F	68	29.1	0.24	0.38
M	69	26.12	0.16	0.11
F	69	29.56	0.28	0.43
M	69	31.1	0.34	0.23
F	72	32.4	0.51	0.68
M	73	30.0	0.25	0.14
M	74	28.98	0.26	0.14
**Mean**	**63.61**	**29.00**	**0.30**	**0.35**
**Maximum**	**74.00**	**35.00**	**0.52**	**0.93**
**Minimum**	**55.00**	**23.66**	**0.16**	**0.11**

**Fig 3:**
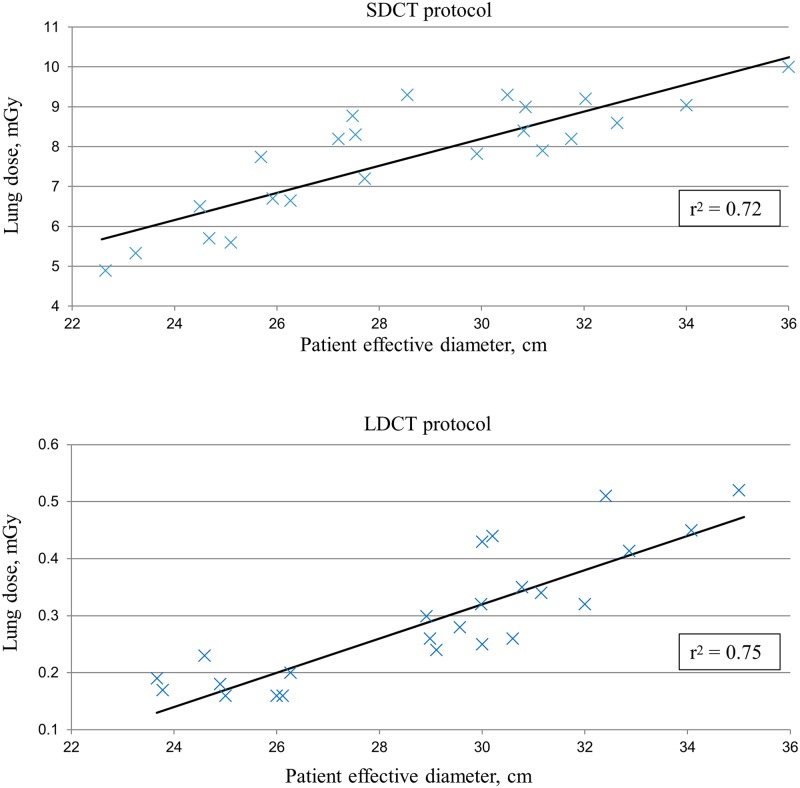
Scatter plots showing the correlation between lung dose and patient effective diameters for standard dose (SDCT) and low-dose CT (LDCT) examinations.

The results for lung dose as a function of patient size (effective diameter) for both protocols are shown in Fig 3. With the SDCT protocol the absolute dose values to the lungs were an order of magnitude higher than those from the LDCT protocol (*p<0*.*001)*. Regression analysis showed a strong linear correlation between patient size and lung dose for both protocols (R^2^ = 0.72 and R^2^ = 0.75 for SDCT and LDCT, respectively).

### Lung cancer risk

**Fig 4:**
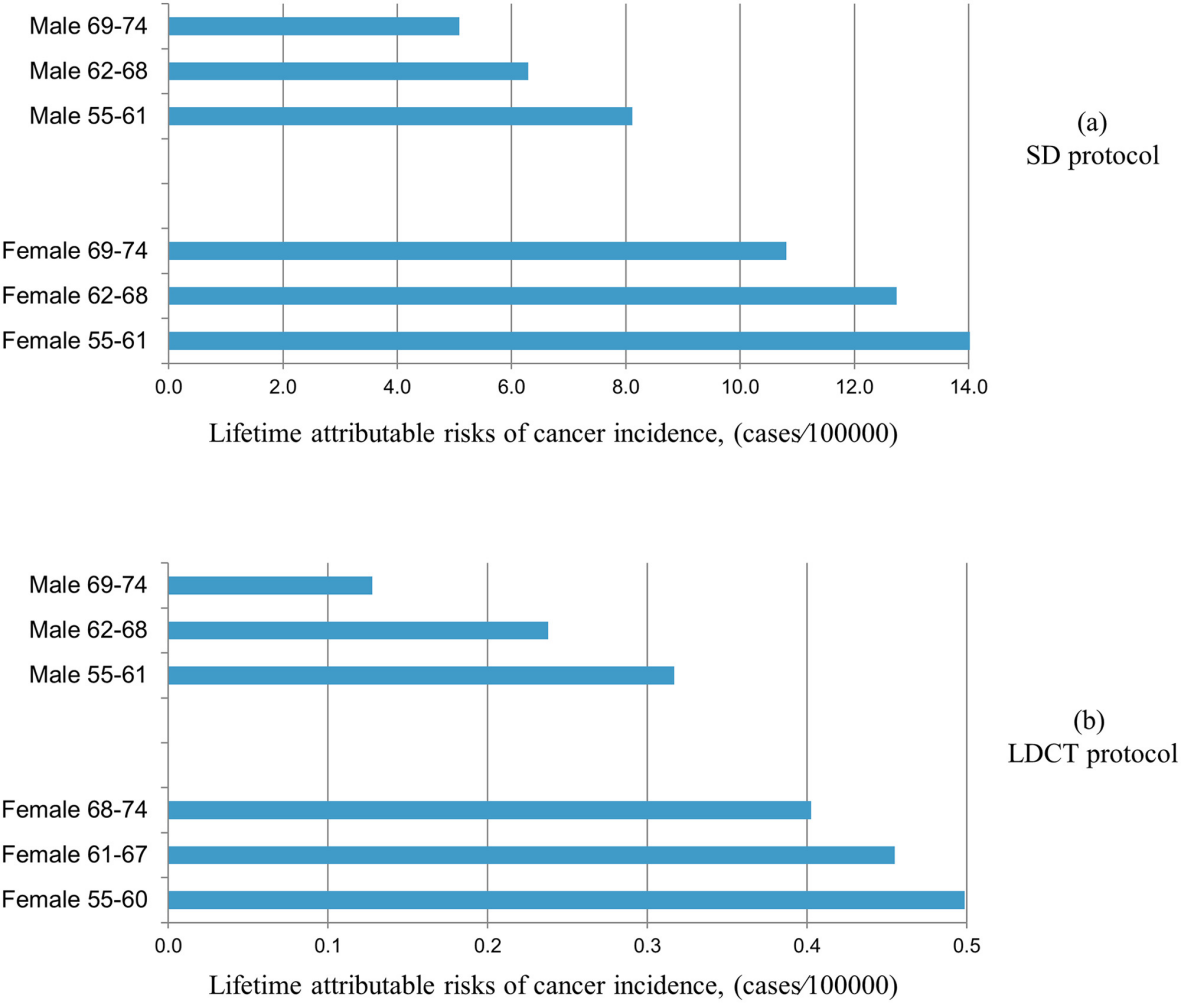
Estimated lifetime attributable risk of cancer as a function of age for males and females from single standard dose (SDCT) and low-dose CT (LDCT) examination.

The average lifetime attributable risk of lung cancer from a single CT examination calculated for male and female patients in separate age groups is shown in Fig 4. The estimated cancer risk values for females were significantly higher than those for males in both LDCT and SDCT protocols (*p* = 0.004, *p* = 0.001) (Table 1). The estimated lifetime attributable risk of lung cancer was significantly lower for the LDCT as compared to the SDCT protocol (*p*<0.001). The risk dropped with higher patient age for both protocols.

## Discussion

In this study we calculated patient-specific lung doses and estimated lifetime attributable cancer risks from chest CT. We compared two different protocols, a SDCT and a recently introduced LDCT protocol with a radiation dose being equivalent of that from chest x-ray, which is reported to be in the range of 0.02mSv for posteroanterior studies and in the range of 0.1mSv for anteroposterior and lateral chest studies [23]. Unlike previous researchers [12–14], we performed dose assessments based on Monte Carlo simulations, taking examination- and patient-specific parameters into account. In a comparison with the rough estimations based on CTDI_vol_ values automatically generated by the scanner, this approach allows not only to estimate the effective dose, but also to calculate the individual patients’ dose to the lungs, and thus, to estimate individual lifetime attributable risks of lung cancer.

This work explicitly focused on lung dose due to the following reason. In contrast to the radiation-associated lifetime attributable cancer risk for most of other organs, which are highest at younger ages, the excess risk for radiation-induced lung cancer is highest in those aged approximately 55 years at exposure [22, 24]. Our study has shown that the dose to the lungs can be significantly reduced when using a LDCT instead of a standard dose protocol. In addition, we could demonstrate a strong linear dependency between lung dose and effective patient diameter for both protocols. This can be explained by the fact that although the CT scanner adjusts the mAs values to the patient size according to an exponential function, the additional adipose tissue in more obese patients serves as natural shielding and thus, the dose for inner organs increases more slowly and can be approximated with a linear function. Similar organ dose dependency on patient size has been already shown by other researchers [25].

The risk assessment performed in the study showed that for both males and females the lifetime attributable cancer risk is decreasing with patient age for both LDCT and SDCT protocols. This can be explained by the fact that risk is not only a function of organ dose but also strongly depends on patient age [22]. Therefore, even if the radiation dose to the lungs for some individuals was higher due to the greater body size, the average risk in older patients (group above 60 years of age) was lower.

Although we found that the lifetime attributable risk for lung cancer from a single LDCT examination was comparatively low, screening would yield higher risk values due to the need for repeated CT examinations. For example, the NLST trial participants received screening with annual low-dose CT for 3 years [26]. In the largest European NELSON trial, current smokers or former smokers who had quit smoking less than 10 years ago underwent one CT examination in years 1, 2, 4 and 6 [27]. The most recent guidelines of American Cancer Society and National Comprehensive Cancer Network recommended LDCT screening for individuals above 55 in each year until the age of 74 [28]. Therefore, the number of CT examination procedures in cancer screening programs varied with the factor 3 up to 25. Given the average lung dose of 7 mGy and 0.3 mGy and corresponding lifetime cancer risk of 8.6 and 0.35 cases per 100’000 population from a single SDCT and LDCT, respectively, as calculated in this study, lung cancer screening as recommended by these societies will be associated with an increase of the attributable lifetime cancer risk up to 215 and 8.7 cases per 100’000 population for SDCT and LDCT, respectively. Thus, it is especially important to keep the radiation dose values from individual CT examinations *as low as reasonably achievable* (the so-called ALARA principle in radiologic imaging with ionizing radiation). By confirming previous studies, we could show that a LDCT protocol applying various radiation dose saving techniques allows for CT lung imaging at a dose equivalent to that of a chest x-ray (0.06mSv).

The following study limitations must be acknowledged. First, the patient CT data used as an input volume for MC simulations was limited by the length of the scan and thus, does not include over-scanning effects in the calculations. However, since in all CT examinations the lung volume was completely covered by the scan range, the effect of over-scanning on the lung dose can be considered negligible [29]. Second, the accuracy of our risk estimations is limited by the uncertainties of current cancer risk models, based on the life-span studies of atomic bomb survivors. Furthermore, the risk coefficients published in the BEIRVII report and used in this study are statistical averages over many individuals of the same gender and similar age. Therefore, care must be taken when interpreting the results of individual patient risk. Nevertheless, the patient-specific risk information as presented in our study represents a step forward beyond effective dose towards personalized patient care Finally, we only investigated one CT scanner from one manufacturer. Since the selective photon shield for single energy is currently a unique technique from one vendor only, results of this study cannot be extrapolated to other systems.

In conclusion, our study determined patient- and examination-specific lung dose values, allowing for individual patient risk assessments, which is mandatory when balancing the benefits and risks from ionizing radiation in the context of lung cancer screening with CT. The lung doses calculated in this study further enhance the need for employing LDCT protocols to lung screening studies for keeping the risk to the general population as low as reasonably possible.
